# PCSK9 targets important for lipid metabolism

**DOI:** 10.1007/s11789-017-0085-0

**Published:** 2017-02-07

**Authors:** Rainer Schulz, Klaus-Dieter Schlüter

**Affiliations:** Department of Physiology, Justus-Liebig-Universität, Aulweg 129, 35392 Giessen, Germany

**Keywords:** Low density lipoprotein (LDL), LDL receptor, Lipoprotein (a)

## Abstract

Ischemic heart disease is the main cause of death worldwide and it is accelerated by increased low-density lipoprotein (LDL) cholesterol (LDL-C) and/or lipoprotein (a) (Lp(a)) concentrations. Proprotein convertase subtilisin/kexin type 9 (PCSK9) alters both LDL-C and in part Lp(a) concentrations through its ability to induce degradation of the LDL receptor (LDLR). PCSK9, however, has additional targets which are potentially involved in lipid metabolism regulation such as the very low density lipoprotein receptor (VLDL), *CD36* (cluster of differentiation 36) and the epithelial cholesterol transporter (NPC1L1) and it affects expression of apolipoprotein B48. The PCSK9 activity is tightly regulated at several levels by factors influencing its transcription, secretion, or by extracellular inactivation and clearance. Many comorbidities (kidney insufficiency, hypothyreoidism, hyperinsulinemia, inflammation) modify PCSK9 expression and release. Two humanized antibodies directed against extracellular PCSK9 received approval by the European and US authorities and additional PCSK9 directed therapeutics (such as silencing RNA) are already in clinical trials. Their results demonstrate a significant reduction in both LDL-C and Lp(a) concentrations – independent of the concomitant medication – and one of them reduced plaque size in high risk cardiovascular patients; results of two ongoing large clinical endpoints studies are awaited. In this review, we summarize and discuss the recent biological data on PCSK9, the regulation of PCSK9, and finally briefly summarize the data of recent clinical studies in the context of lipid metabolism.

## Targets of proprotein convertase subtilisin/kexin type 9 (PCSK9)

Proprotein convertase subtilisin/kexin type 9 (PCSK9) is a member of the proprotein convertase superfamily of serine proteinases also encompassing proprotein convertase 1 (PC1), PC2, PC4, PC5, PC7, furin, paired basic amino acid cleaving enzyme 4 (PACE4), and subtilisin kexin isozyme (SKI)-1 [86]. However, the only PCSK9 substrate identified so far is its own prodomain and autocleavage of ProPCSK9 is a pre-requisite for its subsequent secretion [3]. The cleaved prodomain remains firmly attached in the putative substrate-binding cavity of the catalytic domain thereby preventing PCSK9 from interacting with other substrates.

### Low density lipoprotein (LDL) receptor (LDLR).

**Fig. 1:**
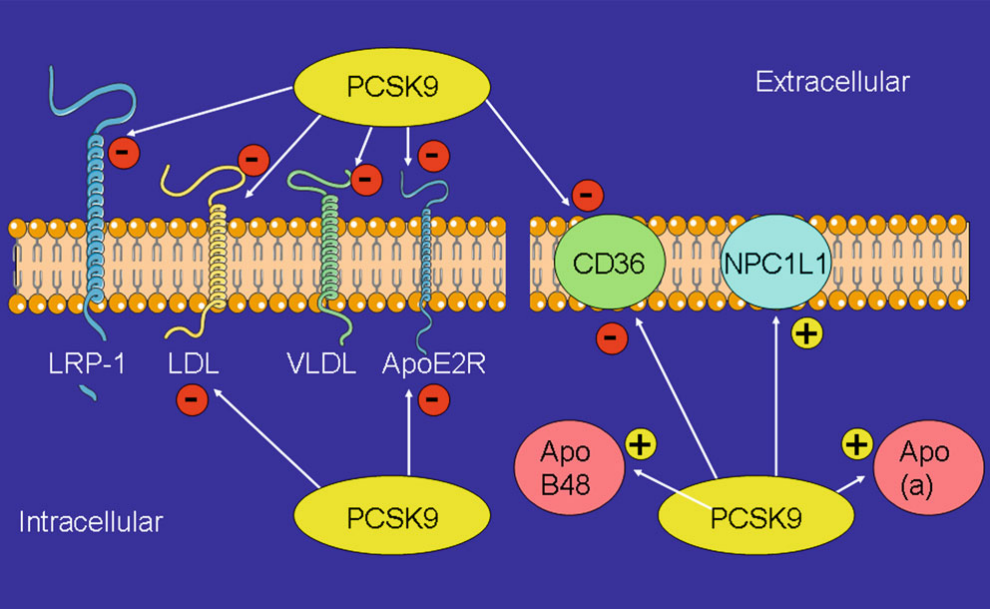
Extra- and intracellular PCSK9 has multiple targets including receptors and transporters, but it also modifies apolipoprotein B48 and apolipoprotein (a) expression (for details, see text). *LRP1* Low density lipoprotein receptor (LDLR) related protein 1, *VLDLR* *very low density lipoprotein receptor*, *ApoER* apolipoprotein E receptor, *CD36* cluster of differentiation 36, *NPC1L1* Niemann-Pick C1-like protein 1, *apo B48* apolipoprotein B48, *apo* *(a)* apolipoprotein (a), – indicates down-regulation or inhibitio, + indicates up-regulation

PCSK9 binds to the extracellular domains of a highly selective subset of transmembrane receptors including LDLR and targets them for degradation in lysosomes by a mechanism that apparently is independent of its proteolytic activity ([83]; Fig. 1). The LDLR binds LDL cholesterol (LDL-C) and removes it from the circulation by mediating its endocytosis via clathrin coated pits. The acidic pH of endosomes causes LDLR to dissociate from LDL-C [56]. LDLR recycles to the cell surface while the LDL-C particle is degraded in lysosomes and recovered cholesterol is used by the cell. LDLR bound to PCSK9 is also endocytosed by a similar clathrin-dependent mechanism but the binding is stronger at acidic pH and instead the entire complex is destined for lysosomal degradation [67, 103]. Accordingly, overexpression of PCSK9 in experiments in mice, hamsters and pigs results in reduced numbers of hepatic LDLR accompanied by marked increases in plasma LDL-C [1, 6, 57, 67]. In contrast, PCSK9 knockout mice are characterized by increased LDLR expression and reduced LDL-C [73].

The absence of a functional LDLR also leads to a marked accumulation of plasma lipoprotein (Lp) (a) in human apolipoprotein (a) transgenic WHHL rabbits suggesting that LDLR may participate in the catabolism of Lp(a) [23]. An effect of LDLR availability on Lp(a) concentration is further supported by cell-association studies in HepG2 cells: Lp(a) cell-association is reduced by co-incubation with LDL-C and PCSK9 suggesting that Lp(a) competes with LDL-C for LDLR binding and internalization. Thus, when LDLR expression is increased – particularly in the setting of low circulating LDL-C – Lp(a) will be reduced [72].

PCSK9 consists of three domains: the N-terminal prodomain followed by the catalytic domain and the C‑terminal domain. The catalytic domain mediates the direct interaction with the epidermal growth factor (EGF) A domain of LDLR as demonstrated using various biochemical methods including crystallography [17, 45, 52].

However, elements of the PCSK9 prodomain appear to have a modulatory effect on LDLR degradation activity as deletion of the sequence stretch 31–58 in the prodomain results in a PCSK9 variant with four to seven fold increased activity. Up to 30% of PCSK9 is bound to LDL-C in mice [27, 96] and normolipidemic subjects [44]. In mice, PCSK9 is also bound to high density lipoprotein (HDL) [27]. For the binding of PCSK9 to LDL-C the amino residues 31–52 of the prodomain are required [44]. The C‑terminal domain on the other hand is required for its ability to induce LDLR degradation as a truncated PCSK9 variant lacking the C‑terminal domain display reduced activity against LDLR whereas the isolated C‑terminal domain alone has no effect on LDLR degradation [105]. The role of the PCSK9 C‑terminal domain is strongly supported by data using a monoclonal antibody directed against the C‑terminal domain which inhibits the ability of PCSK9 to reduce LDL-C uptake in cells and decreases plasma LDL-C in cynomolgus monkeys [63, 78].

Apart from LDL-C, PCSK9 binds to a variety of proteins (for review, see [104]), one of them being annexin A2, which is present in the nucleus, the cytosol and the cell membrane in a variety of cells. The N-terminal repeat R1 of annexin 2 binds to the CHRD region of PCSK9 and inhibits its extracellular LDLR degrading activity [59]. In annexin A2 knockout mice plasma PCSK9 levels are doubled resulting in reduced LDLR expression and an increase in LDL-C [85]; thus annexin A2 is viewed as endogenous inhibitor of PCSK9 [59]. More recently, plasma PCSK9 is found in association with Lp(a) particles in humans with high Lp(a) levels and in mice carrying human Lp(a) [95].

### Very low density lipoprotein receptor (VLDLR).

PCSK9 exerts effects that are independent from the surface expression of LDLR as it also interferes with intracellular transport and degradation of VLDLR [46, 55, 69]. In mice in vivo, circulating PCSK9 – that originates entirely in the liver – regulates VLDLR protein levels in adipose tissue thereby limiting visceral adipogenesis. PCSK9 knockout mice accumulate ≈80% more visceral adipose tissue than wild-type mice and this is associated with adipocyte hypertrophy and increased in vivo fatty acid uptake and ex vivo triglyceride synthesis [74]. VLDLR are implicated also in the removal of Lp(a) from the circulation; in mice deficient for the VLDLR Lp(a) disappearance from the circulation is reduced when compared to control mice [2].

### Other receptors/channels/enzymes affected by PCSK9.

Apart from binding to LDLR and VLDLR, PCSK9 also interacts with other receptors such as the LDLR related protein 1 (LRP1) [10] or the apolipoprotein E receptor (ApoER) [46]. Some interactions of PCSK9 with receptors depend on a EGF-A binding domain (VLDLR) [87] or require the catalytic activity of PCSK9 (LRP1). Apart from interaction with receptors, PCSK9 modifies CD81 on hepatocytes (hepatitis C virus receptor) [47] and CD36 on macrophages (for review, see [89]), intestinal cells [51], adipocytes and in mouse liver [18]. In PCSK9 knockout mice, increased hepatic CD36 expression amplifies the uptake of fatty acid and accumulation of triglycerides and lipid droplets. Peripheral blood mononuclear cells from PCSK9 loss-of-function variant subjects also show significant increases in mRNA levels of CD36 when compared to non-variant controls [30]. PCSK9 knockout mice also present with a significant reduction of lymphatic apolipoprotein B secretion compared to wildtype mice [49]. As the apolipoprotein B concentration is important for the loading of chylomicrons with triglycerides and cholesterol esters [7], PCSK9 deficiency might also protect by reducing postprandial triglyceridemia as measured in PCSK9 knockout mice [49]. Finally, in the intestine, gain-of function (GOF) mutations of PCSK9 up-regulate the cholesterol transporter NPC1L1 (Niemann-Pick C1-like protein 1) and thus cholesterol uptake in a LDLR-independent way [51]. Indeed, the PCSK9 antibody evolocumab has a modest effect on cholesterol synthesis and absorption in humans [68].

## Expression and regulation of PCSK9 expression

### Cell types.

PCSK9 expression is highly restricted both developmentally and in tissues [84]. In adult mice, the highest PCSK9 mRNA levels by far are found in the liver and substantially lower expression is also found in the brain, kidney and small intestine [96]. Under pathophysiological conditions, PCSK9 may be expressed in other cell types as well: in atherosclerotic vascular lesions in humans PCSK9 is detected and released from vascular smooth muscle cells [26]. Also high concentrations of oxidized LDL-C induce PCSK9 expression in endothelial cells which leads to endothelial cell apoptosis; oxidized LDL-C-induced apoptosis is attenuated by silencing RNA directed against PCSK9. Similarly, oxidized LDL-C induced PCSK9 expression in cardiomyocytes [79].

### Hepatic PCSK9 expression.

**Fig. 2:**
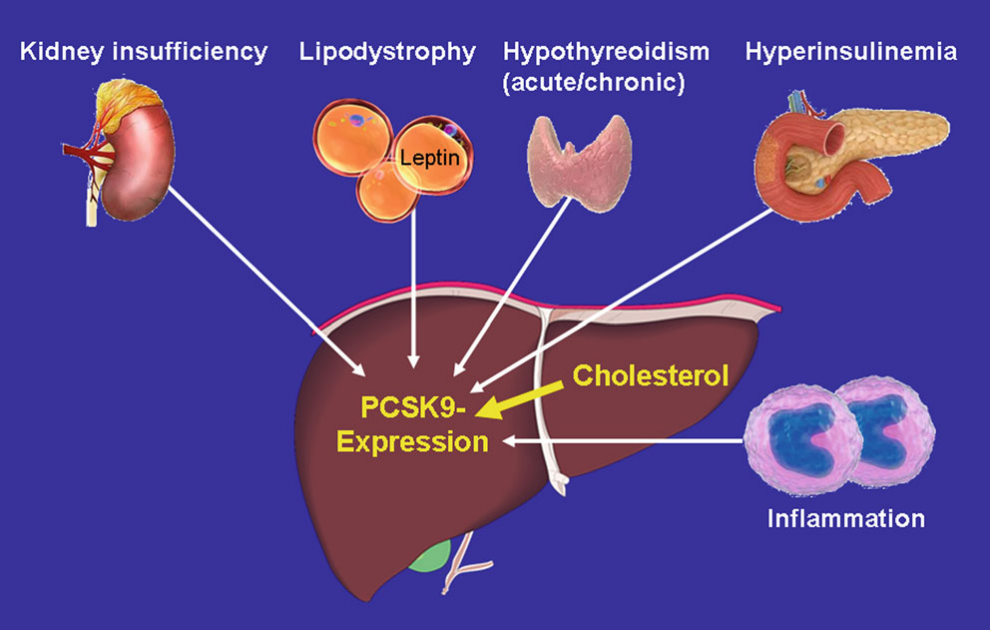
Apart from liver cholesterol content being the most important regulator of PCSK9 expression, co-morbidities influencing hepatic PCSK9 expression and release are indicated

In hepatocytes, up-regulation of PCSK9 by cholesterol depletion or inhibition of intracellular cholesterol synthesis by e. g. statins is explained by a sterol regulatory element [22, 71], which have found to be regulated by sterol-regulatory element binding protein-2 (SREBP-2) and SREBP-1c ([15, 40]; Fig. 2). In close vicinity to the sterol regulatory element, the PCSK9 gene contains a highly conserved hepatocyte nuclear factor 1 (HNF1) binding site and HNF1α has been shown to cooperate with SREBP-2 to regulate PCSK9 expression in HepG2 cells [52] and in the liver [21, 90].

The chaperone heat shock protein 90 kDa β member 1 (= GRP94) residing in the endoplasmic reticulum binds PCSK9 and prevents its ability to sequester LDLR prematurely in the endoplasmic reticulum [70]. Mice lacking GRP94 have highly reduced LDLR level in the liver. The coat protein complex II (COPII) vesicle adaptor protein SEC24A is an endoplasmic reticulum sorting receptor for PCSK9 required for efficient exit of PCSK9 from the endoplasmic reticulum [14]. SEC24A is highly enriched in the liver and SEC24A knockout mice display increased liver LDLR and reduced plasma PCSK9 and LDL-C.

### PCSK9 secretion.

Sortilin is as a high affinity receptor for PCSK9 [35] with an affinity constant in the nanomolar range being close to the actual PCSK9 plasma concentration [48]. Thus, the interaction of sortilin with PCSK9 is up to hundred-fold stronger than the interaction between PCSK9 and the LDLR [27]. Sortilin appears to be involved in intracellular trafficking and release of PCSK9 and sortilin knockout mice display reduced level of circulating PCSK9 and reduced LDL-C [35, 43]. The expression of sortilin is modulated by lifestyle factors – such as diet and exercise – and the plasma sortilin extracellular domain expression correlates with increased body mass index [8].

Several disease states also affect hepatic PCSK9 expression and/or the circulating PCSK9 concentration (Fig. 2):

- Kidney disease increases liver PCSK9 expression and release possibly explaining the hypercholesterolemia observed in patients with chronic kidney disease and nephrotic syndrome [36, 92, 93].
- Lipodystrophy increases PCSK9 concentrations in females and leptin treatment (to cure lipodystrophy) reduces the plasma PCSK9 concentration in parallel with the LDL-C concentration [50].
- Hypothyroidism increases PCSK9 expression in patient with thyroid cancer and hypothyroidism is associated with an increase in LDL-C and apolipoprotein B concentrations [65].
- Hyperinsulinemia increases PCSK9 expression in mice [15] whereas a moderately hyperinsulinemic glucose clamp for 24 h has no effect on PCSK9 plasma concentrations in healthy and type 2 diabetic men [41]. Interestingly, PCSK9 concentration is decreased in patients with type 1 as compared to type 2 diabetes [12], which goes along with data in obese mice with pharmacologically-induced insulin deficiency showing a reduced PCSK9 expression [62].
- Inflammation modulates PCSK9 expression and release. An increase in tumor necrosis factor (TNF) α and in particular activation of the Janus kinase/signal transducer and activator of transcription (JAK/STAT) pathway suppresses PCSK9 transcription in HepG2 cells and in vivo [11]. On the other hand, suppressor of cytokine signaling SOCS3, which is a negative regulator of JAK/STAT, induces PCSK9 expression through activation of SREBP-1 [76]. LRP1, which is regulated by PCSK9 (see above), antagonizes the pro-inflammatory effects of lipopolysaccharides (LPS) and TNFα. While activation of LRP1 depresses nuclear factor kappa-light-chain-enhancer of activated B cells (NFκB) signaling, extracellular LRP1 activates stress activated kinases like p38MAPK [58]. While the activation of LRP1 attenuates LPS-driven inflammation, LPS itself increases the expression of PCSK9 and therefore decreases the hepatic expression of LDLR [24]. Furthermore, the inflammatory cytokine resistin induces PCSK9 expression in human hepatocytes [61]. Intriguingly, the PCSK9 C‑terminal domain displays structural resemblance to resistin suggesting both a structural and functional relationship between cytokine signaling and PCSK9 [37]. Also, C-reactive protein (CRP) increases PCSK9 expression by activating p38 mitogen activated protein kinase (p38MAPK)-HNF1α pathway in HepG2 cells [16].

All the above observations are interesting considering that PCSK9 recently has been described as a critical regulator of the innate immune response and survival following sepsis in both mice and humans [101] (for further review, see [25, 66]).

- Nonalcoholic fatty liver disease (NAFLD), which is associated with cardiovascular disease independently of classic risk factors – increases circulating PCSK9 concentration; the increase in PCSK9 correlates with hepatic fat accumulation and the severity of steatosis, independently of metabolic confounders and liver damage. Thus, modulation of PCSK9 synthesis and release might be involved in NAFLD pathogenesis [75].

### PCSK9 cleavage.

Mature PCSK9 can be detected in plasma and hepatocyte culture supernatant as a 62 kDa active form and a 55 kDa form with reduced activity towards LDLR. The lower molecular form originates from proteolytic processing by the related proprotein convertase furin and to a lesser extent by PC5/6A [4, 38]. Furin cleavage likely primes PCSK9 for further degradation and thereby elimination from the circulation. Recently, matrix metalloproteinase 2 has been found to proteolytically inactivate PCSK9. However, the physiological relevance of this regulatory effect remains to be established [102].

### PCSK9 elimination.

LDLR plays a major role in clearance of plasma PCSK9. In a wildtype mouse iodinated PCSK9 has a half-life of approximately 5 min while this is increased to 15 min in a LDLR knockout mouse [33]. The radioactivity mainly accumulates in the liver but a significant portion is found in the kidney [94]. However, PCSK9 is still relatively rapidly removed from the circulation in the absence of LDLR, suggesting the existence of other clearance mechanisms. This is supported by the fact that individuals homozygous for LDLR inactivating mutations present with markedly increased LDL-C but the PCSK9 levels are not correspondingly high [9]. One obvious clearance mechanism is proteolytic degradation of PCSK9 initiated by furin as described above. Another proposed PCSK9 clearance receptor is amyloid β precursor like protein 2 but this remains to be established in vivo [19, 20].

## Approaches to reduce PCSK9/LDLR interaction and/or PCSK9 synthesis

The interaction of circulating PCSK9 with its potential targets can be attenuated by removing PCSK9 from the circulation (extracellular IgG antibody, monobodies, vaccination) or offering alternative binding partners rather than LDLR (mimetic peptides) (for review see [5, 80]). The synthesis of PCSK9 can be reduced at the level of translation (silencing RNA, oligonucleotides) or its intracellular self-cleavage (mimetic peptides). In this review only treatments with already existing clinical trial data are discussed and data for *PCSK9-antibodies* are summarized in the next chapter.

### PCSK9 silencing RNA.

PCSK9 silencing RNA (siRNA) is formulated in a lipidoid nanoparticle (LNP, Alnylam Pharmaceuticals). Liver-specific silencing of PCSK9 in mice and rats reduces PCSK9 mRNA levels by up to 70% and LDL-C levels by 60%. In non-human primates, a single dose of siRNA targeting PCSK9 results in a rapid, durable, and reversible lowering of plasma PCSK9 and LDL-C lasting for 3 weeks after a single intravenous administration [29]. The siRNA (ALN-PCS) was tested subsequently in a dose-finding study in 32 healthy participants with a LDL-C above 3 mmol/L, ALN-PCS administered intravenously resulted in dose-dependent reductions in plasma PCSK9 and LDL-C levels, with the highest dose conferring 70 and 40% reductions in PCSK9 and LDL-C levels, respectively, an effect which was sustained for 2 to 3 weeks after administration. Alnylam has recently another phase I clinical trial testing subcutaneously administered ALN-PCS demonstrating a sustained reduction of PCSK9 and LDL-C for up to 180 days after a single injection [28].

### PCSK9 antisense oligonucleotides (ASO).

ASOs are short, single-stranded complementary sequences of nucleotides inhibiting protein synthesis by binding to the target mRNA inhibiting protein translation. ASO offer high specificity, but like monocolonal antibodies require intravenous or subcutaneous routes of administration. Two PCSK9-ASOs were initially explored in preclinical trials but development was stopped after a phase I clinical trial (http://www.clinicaltrials.gov; NCT01082562) because of safety concerns [82].

Nucleic acid analogs that contain at least 1 monomer in locked conformation (LNA) provide a higher binding affinity and specificity to the target mRNA [42]. LNA ASO reduces the mRNA and protein levels of PCSK9 with a concomitant increase in LDLR protein levels after transfection in HepG2 and HuH7 cells. In mice, tail vein intravenous administration of LNA ASO reduces the level of PCSK9 mRNA by approximately 60%, an effect lasting more than 16 days. Hepatic LDLR protein levels are significantly up-regulated 3fold for at least 8 days and approximately 2 fold for 16 days [34]. In non-human primates, LNA ASO targeting PCSK9 produced a sustained 50% reduction of LDL-C after a loading dose and four weekly maintenance doses [54]. Although promising preclinical data are available, the first phase I clinical trial testing the efficacy of SPC5001 (Santaris Pharma) – an ASO with locked RNA nucleotides on both ends of the DNA – was terminated for undisclosed reasons (http://www.clinicaltrials.gov; NCT01350960). One potential explanation for study termination relates to renal side effects, since SPC5001 administered subcutaneously in one volunteer increased creatine levels, white blood cells, granular casts, and caused minimal hematuria on urine microscopy. Kidney biopsy showed multifocal tubular necrosis and signs of oligonucleotide accumulation, all changes being reversible upon termination of SPC5001 administration [98].

Thus, several approaches targeting both intra- and extracellular PCSK9 are under development, some of which passed from the pre-clinical into clinical testing (silencing RNA) while others failed (OSA, LNA). Since PCSK9 has multiple intracellular targets [80] one has to see whether long-term results of intra- and extracellular reduction of PCSK9 are advantageous as compared to the extracellular reduction of PCSK9 only.

## Studies of PCSK9-inhibition in patients with high cardiovascular risk

The fully human PCSK9-binding antibodies evolocumab and alirocumab have been approved by the FDA (US Food and Drug Administration) and the EMA (European Medicines Agency) in 2015. Both drugs have been tested in high cardiovascular risk patients on top of maximally tolerated statin treatment.

### LDL-C.

Almost all studies with PCSK9 antibodies report an additional reduction of LDL-C (as well as non-HDL and ApoB) by 50–60% [13]. Even with mild to moderate hepatic impairment, maximum LDL-C reductions in the healthy, mild, and moderate groups were −57%, −70% and −53%, respectively suggesting that the PCSK9 antibody evolocumab can be used without dose adjustment in patients with active liver disease and mild or moderate hepatic impairment [32]. The cholesterol content of several LDL subfractions is reduced and the apolipoproteins CII and CIII and the cholesterol content of very low-density, intermediate-density, and remnant lipoproteins are decreased [97]. High density lipoprotein cholesterol and triglyceride concentrations are not significantly reduced with PCSK9 antibody treatment. Studies such as ODYSSEY CHOICE or LAPLACE confirm that the effect of PCSK9 inhibitor is not reduced but additive in the presences of other oral lipid lowering therapies [91]. The additive effect is consistent with the mechanism of action and the upregulation of PCSK9 serum concentrations by both statins and fibrates [60].

### Lp(a).

Carriers of the PCSK9 R46L (loss-of-function) genetic variant are characterized by low levels of LDL-C and Lp(a) [99], which is a strong cardiovascular risk factor. Clinical studies show that use of the two PCSK9 antibodies alirocumab and evolocumab potently lowers Lp(a) [31, 72, 81], the latter also in patients with type 2 diabetes [77]. Interestingly, PSCK9 inhibition reduces Lp(a) in patient with homozygous familial hypercholesterolemia despite their lack or dysfunction of the LDLR. Therefore the question arises that the regulation of Lp(a) by PCSK9 may be independent of the LDLR. From this perspective, the modulation of VLDLR by PCSK9 appears to be of great interest since Lp(a) clearance by hepatocytes appears to depend on VLDLR expression [39].

**Fig. 3:**
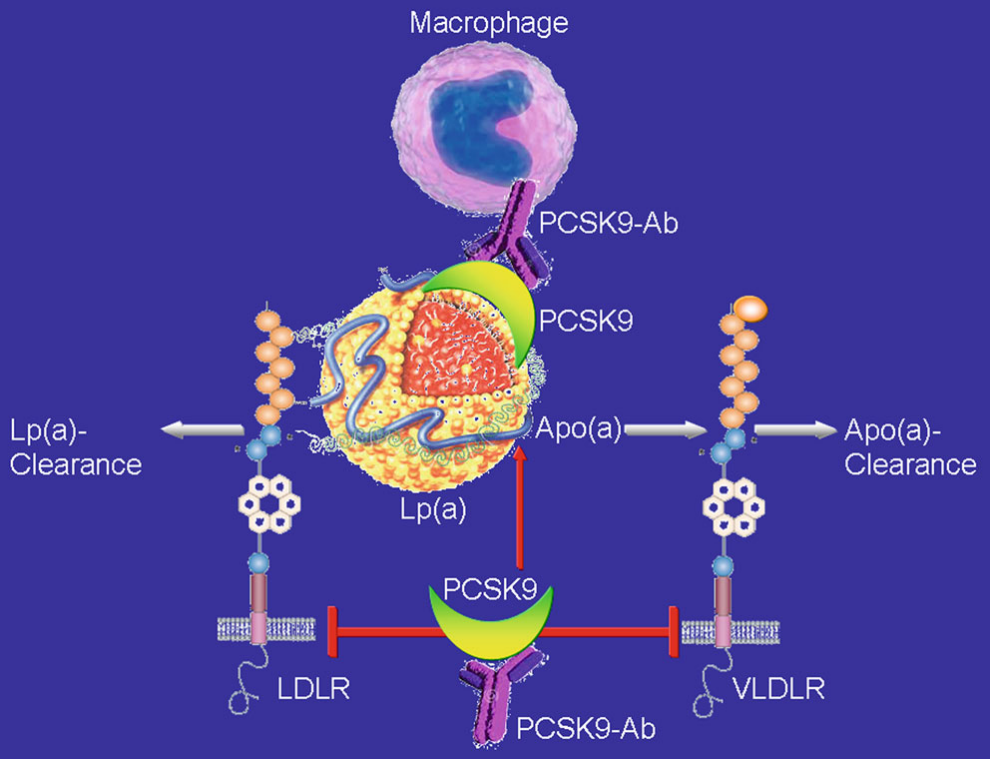
Lipoprotein [Lp](a) clearance occurs via the low density lipoprotein receptor (LDLR) and/or the very low density lipoprotein receptor (VLDLR), both of which are decreased in expression by PCSK9. PCSK9 antibodies by increasing the expression of LDLR and VLDLR thereby might increase Lp(a) clearance. Furthermore, apolipoprotein [(Apo)] (a) is increased by PCSK9 and is reduced by PCSK9 antibody treatment. Finally, PCSK9 binds to Lp(a) and after binding of PCSK9 antibodies, the total construct is taken up by macrophages and thus cleared from the circulation (for further details, see text)

Although the underlying molecular mechanism(s) of reduced Lp(a) concentration by PCSK9 treatment are not fully understood, the following pathways may contribute (Fig. 3):

- The enhanced secretion of Lp(a) from primary human hepatocytes is blunted by PCSK9 inhibition (with alirocumab) [53, 100];
- Reduction of apolipoprotein B or assembly of Lp(a) at outer hepatocyte surface;
- Enhanced removal of Lp(a) in kidney, liver, peripheral tissues, especially after docking of PCSK9 antibodies [88];
- Potential additional receptors for Lp(a) such as docking receptors, sorting receptors sortilin, endocytic receptors (syndecan-1 heparan sulfate proteoglycan);
- Intestinal apolipoprotein metabolism (for review, see [80]).

### Atherosclerosis.

Most recently, the PCSK9 antibody evolocumab met its primary end point of change in percent atheroma volume from baseline to week 78 compared with placebo, as determined by intravascular ultrasound in the GLAGOV trial involving 968 patients with coronary artery disease [64]. LDL-C decreased to 37 mg/dl in the statin + evolocumab group vs 93 mg/dL in the statin group, and this reduction in LDL-C was associated with a reduction in percent atheroma volume for evolocumab and in 2 out of 3 patients a greater percentage of patients demonstrated plaque regression. Post hoc analysis examining the relationship between achieved LDL-C level and change in percent atheroma volume showed a linear reduction down to very low LDL-C levels of 20 mg/dl. The two most important open questions with regard to PCSK9 inhibitor relate to their effects on clinical outcomes and the long-term safety. Two very large outcome trials, FOURIER (~27,000 patients with a history of CVD and at high risk of recurrent events, NCT01764633) and ODYSSEY OUTCOMES (~18,000 patients recently hospitalized for ACS, NCT01663402), are fully recruited and the first outcome results are expected to be reported in March 2017.

## References

[1] Al-Mashhadi RH, Sorensen CB, Kragh PM, Christoffersen C, Mortensen MB, Tolbod LP, Thim T, Du Y, Li J, Liu Y, Moldt B, Schmidt M, Vajta G, Larsen T, Purup S, Bolund L, Nielsen LB, Callesen H, Falk E, Mikkelsen JG, Bentzon JF (2013). Familial hypercholesterolemia and atherosclerosis in cloned minipigs created by DNA transposition of a human PCSK9 gain-of-function mutant. Sci Transl Med.

[2] Argraves KM, Kozarsky KF, Fallon JT, Harpel PC, Strickland DK (1997). The atherogenic lipoprotein Lp(a) is internalized and degraded in a process mediated by the VLDL receptor. J Clin Invest.

[3] Benjannet S, Rhainds D, Essalmani R, Mayne J, Wickham L, Jin W, Asselin MC, Hamelin J, Varret M, Allard D, Trillard M, Abifadel M, Tebon A, Attie AD, Rader DJ, Boileau C, Brissette L, Chretien M, Prat A, Seidah NG (2004). NARC-1/PCSK9 and its natural mutants: zymogen cleavage and effects on the low density lipoprotein (LDL) receptor and LDL cholesterol. J Biol Chem.

[4] Benjannet S, Rhainds D, Hamelin J, Nassoury N, Seidah NG (2006). The proprotein convertase (PC) PCSK9 is inactivated by furin and/or PC5/6A: functional consequences of natural mutations and post-translational modifications. J. Biol. Chem..

[5] Bergeron N, Phan BA, Ding Y, Fong A, Krauss RM (2015). Proprotein convertase subtilisin/kexin type 9 inhibition: a new therapeutic mechanism for reducing cardiovascular disease risk. Circulation.

[6] Bjorklund MM, Hollensen AK, Hagensen MK, Dagnaes-Hansen F, Christoffersen C, Mikkelsen JG, Bentzon JF (2014). Induction of atherosclerosis in mice and hamsters without germline genetic engineering. Circ Res.

[7] Black DD (2007). Development and physiological regulation of intestinal lipid absorption. I. Development of intestinal lipid absorption: cellular events in chylomicron assembly and secretion. Am J Physiol Gastrointest Liver Physiol.

[8] Buttenschon HN, Demontis D, Kaas M, Elfving B, Molgaard S, Gustafsen C, Kaerlev L, Petersen CM, Børglum AD, Mors O, Glerup S (2015). Increased serum levels of sortilin are associated with depression and correlated with BDNF and VEGF. Transl Psychiatry.

[9] Cameron J, Bogsrud MP, Tveten K, Strom TB, Holven K, Berge KE, Leren TP (2012). Serum levels of proprotein convertase subtilisin/kexin type 9 in subjects with familial hypercholesterolemia indicate that proprotein convertase subtilisin/kexin type 9 is cleared from plasma by low-density lipoprotein receptor-independent pathways. Transl Res.

[10] Canuel M, Sun X, Asselin MC, Paramithiotis E, Prat A, Seidah NG (2013). Proprotein convertase subtilisin/kexin type 9 (PCSK9) can mediate degradation of the low density lipoprotein receptor-related protein 1 (LRP-1). PLOS ONE.

[11] Cao A, Wu M, Li H, Liu J (2011). Janus kinase activation by cytokine oncostatin M decreases PCSK9 expression in liver cells. J Lipid Res.

[12] Cariou B, Le Bras M, Langhi C, Le May C, Guyomarc’h-Delasalle B, Krempf M, Costet P (2010). Association between plasma PCSK9 and gamma-glutamyl transferase levels in diabetic patients. Atherosclerosis.

[13] Chapman MJ, Stock JK, Ginsberg NH (2015). PSCK9 inhibitors and cardiovascular disease: heralding a new therapeutic era. Curr Opin Lipidol.

[14] Chen XW, Wang H, Bajaj K, Zhang P, Meng ZX, Ma D, Bai Y, Liu HH, Adams E, Baines A, Yu G, Sartor MA, Zhang B, Yi Z, Lin J, Young SG, Schekman R, Ginsburg D (2013). SEC24A deficiency lowers plasma cholesterol through reduced PCSK9 secretion. Elife.

[15] Costet P, Cariou B, Lambert G, Lalanne F, Lardeux B, Jarnoux AL, Grefhorst A, Staels B, Krempf M (2006). Hepatic PCSK9 expression is regulated by nutritional status via insulin and sterol regulatory element-binding protein 1c. J Biol Chem.

[16] Cui CJ, Li S, Zhu CG, Sun J, Du Y, Zhang Y, Wu NQ, Guo YL, Xu RX, Gao Y, Li JJ (2016). Enhanced pro-protein convertase subtilisin/kexin type 9 expression by C‑reactive protein through p38MAPK-HNF1α pathway in HepG2 cells. J Cell Mol Med.

[17] Cunningham D, Danley DE, Geoghegan DF, Griffor MC, Hawkins JL, Subashi TA, Varghese AH, Ammirati MJ, Culp JS, Hoth LR, Mansour MN, McGrath KM, Seddon AP, Shenolikar S, Stutzman-Engwall KJ, Warren LC, Xia D, Qiu X (2007). Structural and biophysical studies of PCSK9 and its mutants linked to familial hypercholesterolemia. Nat Struct Mol Biol.

[18] Demers A, Samami S, Lauzier B, Des Rosiers C, Ngo Sock ET, Ong H, Mayer G (2015). PCSK9 induces CD36 degradation and affects long-chain fatty acid uptake and triglyceride metabolism in adipocytes and in mouse liver. Arterioscler Thromb Vasc Biol.

[19] DeVay RM, Shelton DL, Liang H (2013). Characterization of proprotein convertase subtilisin/kexin type 9 (PCSK9) trafficking reveals a novel lysosomal targeting mechanism via amyloid precursor-like protein 2 (APLP2). J Biol Chem.

[20] DeVay RM, Yamamoto L, Shelton DL, Liang H (2015). Common Proprotein Convertase Subtilisin/Kexin type 9 (PCSK9) epitopes mediate multiple routes for internalization and function. PLOS ONE.

[21] Dong B, Wu M, Li H, Kraemer FB, Adeli K, Seidah NG, Park SW, Liu J (2010). Strong induction of PCSK9 gene expression through HNF1alpha and SREBP2: mechanism for the resistance of LDL-cholesterol lowering effect of statins in dyslipidemic hamsters. J Lipid Res.

[22] Dubuc G, Chamberland A, Wassef H, Davignon J, Seidah NG, Bernier L, Prat A (2004). Statins upregulate PCSK9, the gene encoding the proprotein convertase neural apoptosis-regulated convertase-1 implicated in familial hypercholesterolemia. Arterioscler Thromb Vasc Biol.

[23] Fan J, Challah M, Shimoyamada H, Shiomi M, Marcovina S, Watanabe T (2010). Defects of the LDL receptor in WHHL transgenic rabbits lead to a marked accumulation of plasma lipoprotein[a]. J Lipid Res.

[24] Feingold KR, Moser AH, Shigenaga JK, Patzek SM, Grunfeld C (2008). Inflammation stimulates the expression of PCSK9. Biochem Biophys Res Commun.

[25] Ferri N, Ruscica M (2016). Proprotein convertase subtilisin/kexin type 9 (PCSK9) and metabolic syndrome: insights on insulin resistance, inflammation, and atherogenic dyslipidemia. Endocrine.

[26] Ferri N, Tibolla G, Pirillo A, Cipollone F, Mezzetti A, Pacia S, Corsini A, Catapano AL (2012). Proprotein convertase subtilisin kexin type 9 (PCSK9) secreted by cultured smooth muscle cells reduces macrophages LDLR levels. Atherosclerosis.

[27] Fisher TS, Lo SP, Pandit S, Mattu M, Santoro JC, Wisniewski D, Cummings RT, Calzetta A, Cubbon RM, Fischer PA, Tarachandani A, De FR, Wright SD, Sparrow CP, Carfi A, Sitlani A (2007). Effects of pH and low density lipoprotein (LDL) on PCSK9-dependent LDL receptor regulation. J Biol Chem.

[28] Fitzgerald K, White S, Borodovsky A, Bettencourt BR, Strahs A, Clausen V, Wijngaard P, Horton JD, Taubel J, Brooks A, Fernando C, Kauffman RS, Kallend D, Vaishnaw A, Simon A (2016). A highly durable RNAi therapeutic inhibitor of PCSK9. N Eng J Med.

[29] Frank-Kamenetsky M, Grefhorst A, Anderson NN, Racie TS, Bramlage B, Akinc A, Butler D, Charisse K, Dorkin R, Fan Y, Gamba-Vitalo C, Hadwiger P, Jayaraman M, John M, Jayaprakash KN, Maier M, Nechev L, Rajeev KG, Read T, Rohl I, Soutschek J, Tan P, Wong J, Wang G, Zimmermann T, de Fougerolles A, Vornlocher H-P, Langer R, Anderson DG, Manoharan M, Koteliansky V, Horton JD, Fitzgerald K (2008). Therapeutic RNAi targeting PCSK9 acutely lowers plasma cholesterol in rodents and LDL cholesterol in nonhuman primates. Proc Natl Acad Sci USA.

[30] Gagnon A, Ooi TC, Cousins M, Favreau C, Henry K, Landry A, Sorisky A (2016). The anti-adipogenic effect of peripheral blood mononuclear cells is absent with PCSK9 loss-of-function variants. Obesity (Silver Spring).

[31] Gaudet D, Watts GF, Robinson JG, Minini P, Sasiela WJ, Edelberg J, Louie MJ, Raal FJ (2017). Effect of alirocumab on lipoprotein(a) over ≥1.5 years (from the phase 3 ODYSSEY program). Am J Cardiol.

[32] Gibbs JP, Slatter JG, Egbuna O, Geller M, Hamilton L, Dias CS, Xu RY, Johnson J, Wasserman SM, Emery MG (2016). Evaluation of evolocumab (AMG 145), a fully human anti-PCSK9 IgG2 monoclonal antibody, in subjects with hepatic impairment. J Clin Pharmacol.

[33] Grefhorst A, McNutt MC, Lagace TA, Horton JD (2008). Plasma PCSK9 preferentially reduces liver LDL receptors in mice. J Lipid Res.

[34] Gupta N, Fisker N, Asselin MC, Lindholm M, Rosenbohm C, Orum H, Elmen J, Seidah NG (2010). A locked nucleic acid antisense oligonucleotide (LNA) silences PCSK9 and enhances LDLR expression in vitro and in vivo. PLOS ONE.

[35] Gustafsen C, Kjolby M, Nyegaard M, Mattheisen M, Lundhede J, Buttenschon H, Mors O, Bentzon JF, Madsen P, Nykjaer A, Glerup S (2014). The hypercholesterolemia-risk gene SORT1 facilitates PCSK9 secretion. Cell Metab.

[36] Haas ME, Levenson AE, Sun X, Liao WH, Rutkowski JM, de Ferranti SD, Schumacher VA, Scherer PE, Salant DJ, Biddinger SB (2016). The role of proprotein convertase subtilisin/kexin type 9 in nephrotic syndrome-associated hypercholesterolemia. Circulation.

[37] Hampton EN, Knuth MW, Li J, Harris JL, Leslie SA, Spraggon G (2007). The self-inhibited structure of full-length PCSK9 at 1.9 A reveals structural homology with resistin within the C‑terminal domain. Proc Natl Acad Sci USA.

[38] Han B, Eacho PI, Knierman MD, Troutt JS, Konrad RJ, Yu X, Schroeder KM (2014). Isolation and characterization of the circulating truncated form of PCSK9. J Lipid Res.

[39] Hoover-Plow J, Huang M (2013). Lipoprotein(a) metabolism: potential sites for therapeutic targets. Metabolism.

[40] Jeong HJ, Lee HS, Kim KS, Kim YK, Yoon D, Park SW (2008). Sterol-dependent regulation of proprotein convertase subtilisin/kexin type 9 expression by sterol regulatory element-binding protein-2. J Lipid Res.

[41] Kapelle PJ, Lambert G, Dullaart RP (2011). Plasma proprotein convertase subtilisin-kexin type 9 does not change during 24 h insulin infusion in healthy subjects and type 2 diabetic patients. Atherosclerosis.

[42] Kauppinen S, Vester B, Wengel J (2005). Locked nucleic acid (LNA): high affinity targeting of RNA for diagnosis and therapeutics. Drug Discov Today Technol.

[43] Kjolby M, Nielsen MS, Petersen CM (2015). Sortilin, encoded by the cardiovascular risk gene SORT1, and its suggested functions in cardiovascular disease. Curr Atheroscler Rep.

[44] Kosenko T, Golder M, Leblond G, Weng W, Lagace TA (2013). Low density lipoprotein binds to proprotein convertase subtilisin/kexin type-9 (PCSK9) in human plasma and inhibits PCSK9-mediated low density lipoprotein receptor degradation. J Biol Chem.

[45] Kwon HJ, Lagace TA, McNutt MC, Horton JD, Deisenhofer J (2008). Molecular basis for LDL receptor recognition by PCSK9. Proc Natl Acad Sci USA.

[46] Kysenius K, Muggalla P, Matlik K, Arumae U, Huttunen HJ (2012). PCSK9 regulates neuronal apoptosis by adjusting ApoER2 levels and signaling. Cell Mol Life Sci.

[47] Labonte P, Begley S, Guevin C, Asselin MC, Nassoury N, Mayer G, Prat A, Seidah NG (2009). PCSK9 impedes hepatitis C virus infection in vitro and modulates liver CD81 expression. Hepatology.

[48] Lakoski SG, Lagace TA, Cohen JC, Horton JD, Hobbs HH (2009). Genetic and metabolic determinants of plasma PCSK9 levels. J Clin Endocrinol Metab.

[49] Le May C, Kourimate S, Langhi C, Chétiveaux M, Jarry A, Comera C, Collet X, Kuipers F, Krempf M, Cariou B, Costet P (2009). Proprotein convertase subtilisin kexin type 9 null mice are protected from postprandial triglyceridemia. Arterioscler Thromb Vasc Biol.

[50] Levenson AE, Haas ME, Miao J, Brown RJ, de Ferranti SD, Muniyappa R, Biddinger SB (2016). Effect of leptin replacement on PCSK9 in ob/ob mice and female lipodystrophic patients. Endocrinology.

[51] Levy E, Ben Djoudi Ouadda A, Spahis S, Sane AT, Garofalo C, Grenier É, Emonnot L, Yara S, Couture P, Beaulieu JF, Ménard D, Seidah NG, Elchebly M (2013). PCSK9 plays a significant role in cholesterol homeostasis and lipid transport in intestinal epithelial cells. Atherosclerosis.

[52] Li H, Dong B, Park SW, Lee H-S, Chen W, Liu J (2009). Hepatocyte nuclear factor 1alpha plays a critical role in PCSK9 gene transcription and regulation by the natural hypocholesterolemic compound berberine. J Biol Chem.

[53] Libby P (2016). Lipoprotein (a): a frustrating final frontier in lipid management?. JACC Basic Transl Sci.

[54] Lindholm MW, Elmen J, Fisker N, Hansen HF, Persson R, Moller MR, Rosenbohm C, Orum H, Straarup EM, Koch T (2012). PCSK9 LNA antisense oligonucleotides induce sustained reduction of LDL cholesterol in nonhuman primates. Mol. Ther..

[55] Liu M, Wu G, Baysarowich J, Kavana M, Addona GH, Bierilo KK, Mudgett JS, Pavlovic G, Sitlani A, Renger JJ, Hubbard BK, Fisher TS, Zerbinatti CV (2010). PCSK9 is not involved in the degradation of LDL receptors and BACE1 in the adult mouse brain. J Lipid Res.

[56] Lo Surdo P, Bottomley MJ, Calzetta A, Settembre EC, Cirillo A, Pandit S, Ni YG, Hubbard B, Sitlani A, Carfí A (2011). Mechanistic implications for LDL receptor degradation from the PCSK9/LDLR structure at neutral pH. EMBO Rep..

[57] Maxwell KN, Breslow JL (2004). Adenoviral-mediated expression of Pcsk9 in mice results in a low-density lipoprotein receptor knockout phenotype. Proc Natl Acad Sci USA.

[58] May P (2013). The low-density lipoprotein receptor-related protein 1 in inflammation. Curr Opin Lipidol.

[59] Mayer G, Poirier S, Seidah NG (2008). Annexin A2 is a C-terminal PCSK9-binding protein that regulates endogenous low density lipoprotein receptor levels. J Biol Chem.

[60] Mayne J, Dewpura T, Raymond A, Cousins M, Chaplin A, Lahey KA, Lahaye SA, Mbikay M, Ooi TC, Chretien M (2008). Plasma PCSK9 levels are significantly modified by statins and fibrates in humans. Lipids Health Dis.

[61] Melone M, Wilsie L, Palyha O, Strack A, Rashid S (2012). Discovery of a new role of human resistin in hepatocyte low-density lipoprotein receptor suppression mediated in part by proprotein convertase subtilisin/kexin type 9. J Am Coll Cardiol.

[62] Miao J, Manthena PV, Haas ME, Ling AV, Shin DJ, Graham MJ, Crooke RM, Liu J, Biddinger SB (2015). Role of insulin in the regulation of proprotein convertase subtilisin/kexin type 9. Arterioscler Thromb Vasc Biol.

[63] Ni YG, Condra JH, Orsatti L, Shen X, Di Marco S, Pandit S, Bottomley MJ, Ruggeri L, Cummings RT, Cubbon RM, Santoro JC, Ehrhardt A, Lewis D, Fisher TS, Ha S, Njimoluh L, Wood DD, Hammond HA, Wisniewski D, Volpari C, Noto A, Lo Surdo P, Hubbard B, Carfí A, Sitlani A (2010). A proprotein convertase subtilisin-like/kexin type 9 (PCSK9) C‑terminal domain antibody antigen-binding fragment inhibits PCSK9 internalization and restores low density lipoprotein uptake. J Biol Chem.

[64] Nicholls S, Puri R, Anderson T, Ballantyne CM, Cho L, Kastelein JJP, Koenig W, Somaratne R, Kassahun H, Yang J, Wasserman SM, Scott R, Ungi I, Podolec J, Ophuis AO, Cornel JH, Borgman M, Brenann DM, Nissen SE (2016). Effect of evolocumab on progression of coronary disease in statin-treated patients: the GLAGOV randomized clinical trial. JAMA.

[65] Ozkan C, Akturk M, Altinova AE, Cerit ET, Gulbahar O, Yalcin MM, Cakir N, Balos Toruner F (2015). Proprotein convertase subtilisin/kexin type 9 (PCSK9), soluble lectin-like oxidized LDL receptor 1 (sLOX-1) and ankle brachial index in patients with differentiated thyroid cancer. Endocr J.

[66] Paciullo F, Fallarino F, Bianconi V, Mannarino MR, Sahebkar A, Pirro M (2017). PCSK9 at the crossroad of cholesterol metabolism and immune function during infections. J Cell Physiol.

[67] Park SW, Moon YA, Horton JD (2014). Post-transcriptional regulation of low density lipoprotein receptor protein by proprotein convertase subtilisin/kexin type 9a in mouse liver. J Biol Chem.

[68] Peach M, Xu R, Fitzpatrick D, Hamilton L, Somaratne R, Scott R, Wasserman SM, Djedjos CS (2016). Effect of evolocumab on cholesterol synthesis and absorption. J Lipid Res.

[69] Poirier S, Mayer G, Benjannet S, Bergeron E, Marcinkiewicz J, Nassoury N, Mayer H, Nimpf J, Prat A, Seidah NG (2008). The proprotein convertase PCSK9 induces the degradation of low density lipoprotein receptor (LDLR) and its closest family members VLDLR and ApoER2. J Biol Chem.

[70] Poirier S, Mamarbachi M, Chen WT, Lee AS, Mayer G (2015). GRP94 regulates circulating cholesterol levels through blockade of PCSK9-induced LDLR degradation. Cell Rep.

[71] Raal FJ, Giugliano RP, Sabatine MS, Koren MJ, Blom D, Seidah NG, Honarpour N, Lira A, Xue A, Chiruvolu P, Jackson S, Di M, Peach M, Somaratne R, Wasserman SM, Rashid S, Curtis DE, Garuti R, Anderson NN, Bashmakov Y, Ho YK, Hammer RE, Moon YA, Horton JD (2005). Decreased plasma cholesterol and hypersensitivity to statins in mice lacking PCSK9. Proc Natl Acad Sci USA.

[72] Raal FJ, Giugliano RP, Sabatine MS, Koren MJ, Blom D, Seidah NG, Honarpour N, Lira A, Xue A, Chiruvolu P, Jackson S, Di M, Peach M, Somaratne R, Wasserman SM, Scott R, Stein EA (2016). PCSK9 inhibition-mediated reduction in Lp(a) with evolocumab: an analysis of 10 clinical trials and the LDL receptor’s role. J Lipid Res.

[73] Rashid S, Curtis DE, Garuti R, Anderson NN, Bashmakov Y, Ho YK, Hammer RE, Moon YA, Horton JD (2005). Decreased plasma cholesterol and hypersensitivity to statins in mice lacking PCSK9. Proc Natl Acad Sci USA.

[74] Roubtsova A, Munkonda MN, Awan Z, Marcinkiewicz J, Chamberland A, Lazure C, Cianflone K, Seidah NG, Prat A (2011). Circulating proprotein convertase subtilisin/kexin 9 (PCSK9) regulates VLDLR protein and triglyceride accumulation in visceral adipose tissue. Arterioscler Thromb Vasc Biol.

[75] Ruscica M, Ferri N, Macchi C, Meroni M, Lanti C, Ricci C, Maggioni M, Fracanzani AL, Badiali S, Fargion S, Magni P, Valenti L, Dongiovanni P (2016). Liver fat accumulation is associated with circulating PCSK9. Ann Med.

[76] Ruscica M, Ricci C, Macchi C, Magni P, Cristofani R, Liu J, Corsini A, Ferri N (2016). Suppressor of cytokine signaling-3 (SOCS-3) induces proprotein convertase subtilisin kexin type 9 (PCSK9) expression in hepatic HepG2 cell line. J Biol Chem.

[77] Sattar N, Preiss D, Robinson JG, Djedjos CS, Elliott M, Somaratne R, Wasserman SM, Raal FJ (2016). Lipid-lowering efficacy of the PCSK9 inhibitor evolocumab (AMG 145) in patients with type 2 diabetes: a meta-analysis of individual patient data. Lancet Diabetes Endocrinol.

[78] Schiele F, Park J, Redemann N, Luippold G, Nar H (2014). An antibody against the C‑terminal domain of PCSK9 lowers LDL cholesterol levels in vivo. J Mol Biol.

[79] Schlüter K-D, Weber M, Schreckenberg R, Schulz R (2016) ox-LDL and Angiotensin: Cooperative effects via induction of PCSK9 in cardiomyocytes. Clin Res Cardiol 105, Suppl 1, V1278

[80] Schulz R, Schlüter K-D, Laufs U (2015). Molecular and cellular function of the proprotein convertase subtilisin/kexin type 9 (PCSK9). Basic Res Cardiol.

[81] Scott R, Stein EA (2016). PCSK9 inhibition-mediated reduction in Lp(a) with evolocumab: an analysis of 10 clinical trials and the LDL receptor’s role. J Lipid Res.

[82] Sehgal A, Vaishnaw A, Fitzgerald K (2013). Liver as a target for oligonucleotide therapeutics. J Hepatol.

[83] Seidah NG, Awan Z, Chretien M, Mbikay M (2014). PCSK9: a key modulator of cardiovascular health. Circ Res.

[84] Seidah NG, Benjannet S, Wickham L, Marcinkiewicz J, Jasmin SB, Stifani S, Basak A, Prat A, Chretien M (2003). The secretory proprotein convertase neural apoptosis-regulated convertase 1 (NARC-1): liver regeneration and neuronal differentiation. Proc Natl Acad Sci USA.

[85] Seidah NG, Poirier S, Denis M, Parker R, Miao B, Mapelli C, Prat A, Wassef H, Davignon J, Hajjar KA, Mayer G (2012). Annexin A2 is a natural extrahepatic inhibitor of the PCSK9-induced LDL receptor degradation. PLOS ONE.

[86] Seidah NG, Prat A (2012). The biology and therapeutic targeting of the proprotein convertases. Nat Rev Drug Discov.

[87] Shan L, Pang L, Zhang R, Murgolo NJ, Lan H, Hedrick JA (2008). PCSK9 binds to multiple receptors and can be functionally inhibited by an EGF-A peptide. Biochem Biophys Res Commun.

[88] Shapiro MD, Fazio S (2016). From lipids to inflammation: new approaches to reducing atherosclerotic risk. Circ Res.

[89] Shen L, Peng HC, Nees SN, Zhao SP, Xu DY (2013). Proprotein convertase subtilisin/kexin type 9 potentially influences cholesterol uptake in macrophages and reverse cholesterol transport. FEBS Lett.

[90] Shende VR, Wu M, Singh AB, Dong B, Can CF, Liu J (2015). Reduction of PCSK9 and LDL-C levels by liver-specific knockdown of HNF1alpha in normolipemic mice. J Lipid Res.

[91] Stroes ES, Colquhoun D, Sullivan D, Civeira F, Rosenson RS, Watts GF, Bruckert E, Cho L, Dent R, Knusel B, Xue A, Scott R, Wasserman SM, Rocco M (2014). Anti-PCSK-9 antibody effectively lowers cholesterol in patients with statin intolerance. J Am Coll Cardiol.

[92] Sucajtys-Szulc E, Szolkiewicz M, Swierczynski J, Rutkowski B (2016). Up-regulation of Hnf1α gene expression in the liver of rats with experimentally induced chronic renal failure – a possible link between circulating PCSK9 and triacylglycerol concentrations. Atherosclerosis.

[93] Sucajtys-Szulc E, Szolkiewicz M, Swierczynski J, Rutkowski B (2016). Up-regulation of liver Pcsk9 gene expression as a possible cause of hypercholesterolemia in experimental chronic renal failure. Mol Cell Biochem.

[94] Tavori H, Fan D, Blakemore JL, Yancey PG, Ding L, Linton MF, Fazio S (2013). Serum protease converatse subtilisin/kexin type 9 and cell surface low-density lipoprotein receptor: evidence for a reciprocal regulation. Circulation.

[95] Tavori H, Christian D, Minnier J, Plubell D, Shapiro MD, Yeang C, Giunzioni I, Croyal M, Duell PB, Lambert G, Tsimikas S, Fazio S (2016). PCSK9 association with lipoprotein(a). Circ Res.

[96] Tavori H, Fan D, Blakemore JL, Yancey PG, Ding L, Linton MF, Fazio S (2013). Serum proprotein convertase subtilisin/kexin type 9 and cell surface low-density lipoprotein receptor: evidence for a reciprocal regulation. Circulation.

[97] Toth PP, Hamon SC, Jones SR, Martin SS, Joshi PH, Kulkarni KR, Banerjee P, Hanotin C, Roth EM, McKenney JM (2016). Effect of alirocumab on specific lipoprotein non-high-density lipoprotein cholesterol and subfractions as measured by the vertical auto profile method: analysis of 3 randomized trials versus placebo. Lipids Health Dis.

[98] Van Poelgeest EP, Hodges MR, Moerland M, Tesiier Y, Levin AA, Persson R, Lindholm MW, Dumong Erichsen K, Orum H, Cohen AF, Burggraaf J (2015). Antisense-mediated reduction of proprotein convertase subtilisin/kexin type 9 (PCSK9): a first-in-human randomized, placebo-controlled trial. Br J Clin Pharmacol.

[99] Verbeek R, Boyer M, Boekholdt SM, Hovingh GK, Kastelein JJ, Wareham N, Khaw KT, Arsenault BJ (2017). Carriers of the PCSK9 R46L variant are characterized by an antiatherogenic lipoprotein profile assessed by nuclear magnetic resonance spectroscopy-brief report. Arterioscler Thromb Vasc Biol.

[100] Villard EF, Thedrez A, Blankenstein J, Croyal M, Tran TTT, Poirier B, Le Bail JC, Illiano S, Nobécourt E, Krempf M, Blom DJ, Marais AD, Janiak P, Muslin AJ, Guillot E, Lambert G (2016). PCSK9 modulates the secretion but not the cellular uptake of lipoprotein(a) ex vivo: an effect blunted by alirocumab. JACC Basic Transl Sci.

[101] Walley KR, Thain KR, Russell JA, Reilly MP, Meyer NJ, Ferguson JF, Christie JD, Nakada TA, Fjell CD, Thair SA, Cirstea MS, Boyd JH (2014). PCSK9 is a critical regulator of the innate immune response and septic shock outcome. Sci Transl Med.

[102] Wang X, Berry E, Hernandez-Anzaldo S, Sun D, Adijiang A, Li L, Zhang D, Fernandez-Patron C (2015). MMP-2 inhibits PCSK9-induced degradation of the LDL receptor in Hepa1-c1c7 cells. FEBS Lett.

[103] Wang Y, Huang Y, Hobbs HH, Cohen JC (2012). Molecular characterization of proprotein convertase subtilisin/kexin type 9‑mediated degradation of the LDLR. J Lipid Res.

[104] Xu W, Liu L, Hornby D (2012). c-IAP1 binds and processes PCSK9 protein: linking the c‑IAP1 in a TNF-alpha pathway to PCSK9-mediated LDLR degradation pathway. Molecules.

[105] Zhang DW, Garuti R, Tang WJ, Cohen JC, Hobbs HH (2008). Structural requirements for PCSK9-mediated degradation of the low-density lipoprotein receptor. Proc Natl Acad Sci USA.

